# Applications of Additively Manufactured Tools in Abrasive Machining—A Literature Review

**DOI:** 10.3390/ma14051318

**Published:** 2021-03-09

**Authors:** Mariusz Deja, Dawid Zieliński, Aini Zuhra Abdul Kadir, Siti Nur Humaira

**Affiliations:** 1 Department of Manufacturing and Production Engineering, Faculty of Mechanical Engineering and Ship Technology, Institute of Machine and Materials Technology, Gdańsk University of Technology, G. Narutowicza Str. 11/12, 80-233 Gdańsk, Poland; dawid.zielinski@pg.edu.pl; 2 School of Mechanical Engineering, Faculty of Engineering, Universiti Teknologi Malaysia, Skudai 81310, Johor Bahru, Malaysia; ainizuhra@utm.my (A.Z.A.K.); myrah92@yahoo.com (S.N.H.)

**Keywords:** additive manufacturing, abrasive processes, abrasive tools, rapid prototyping, rapid tooling

## Abstract

High requirements imposed by the competitive industrial environment determine the development directions of applied manufacturing methods. 3D printing technology, also known as additive manufacturing (AM), currently being one of the most dynamically developing production methods, is increasingly used in many different areas of industry. Nowadays, apart from the possibility of making prototypes of future products, AM is also used to produce fully functional machine parts, which is known as Rapid Manufacturing and also Rapid Tooling. Rapid Manufacturing refers to the ability of the software automation to rapidly accelerate the manufacturing process, while Rapid Tooling means that a tool is involved in order to accelerate the process. Abrasive processes are widely used in many industries, especially for machining hard and brittle materials such as advanced ceramics. This paper presents a review on advances and trends in contemporary abrasive machining related to the application of innovative 3D printed abrasive tools. Examples of abrasive tools made with the use of currently leading AM methods and their impact on the obtained machining results were indicated. The analyzed research works indicate the great potential and usefulness of the new constructions of the abrasive tools made by incremental technologies. Furthermore, the potential and limitations of currently used 3D printed abrasive tools, as well as the directions of their further development are indicated.

## 1. Introduction

**Figure 1:**
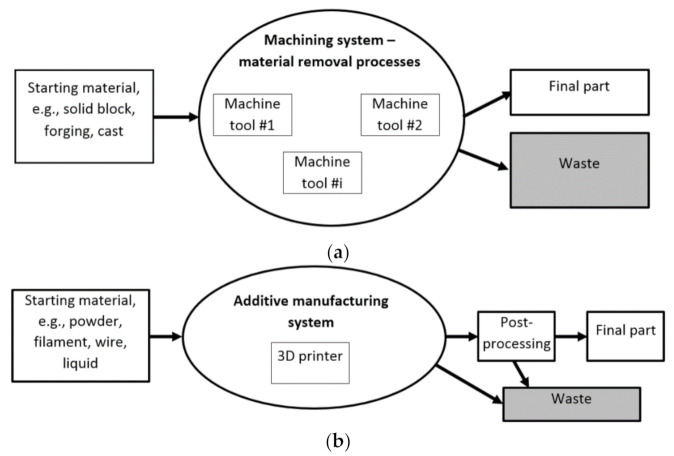
Schematic diagrams of the subtractive manufacturing process (**a**) and the additive manufacturing process (**b**).

Nowadays, the proper selection of an appropriate manufacturing method, machine tools and tools is one of the most crucial decisions in the product development cycle [1,2]. Additive manufacturing (AM) systems can be defined as a new generation of Flexible Manufacturing Systems (FMS) in which a variety of different products with different materials can be flexibly produced using the same machines [3]. The unquestionable advantages of additive techniques, related, among other things, to the possibility of having design freedom allowing complex shapes to be produced, improving the mechanical strength properties of the products, and shortening the lead times, still come with a limited number of available materials and the necessity of post-processing to improve the surface finish and dimensional accuracy. Nevertheless, advanced materials, including composite materials with reinforcement phases, can be used for making composite and complex products working under heavy-duty operating conditions. Other than that, composite materials can also be used to improve the dimensional accuracy of the printed parts. In a study by Deja et al. [4], the Analytic Hierarchy Process (AHP) technique was used as a decisive support method to compare a standard subtractive method with additive manufacturing for the fabrication of offshore machinery components. The obtained results showed that under specific technical requirements and production resources, CNC milling proved to be the most appropriate method for the fabrication of the analyzed part. This could also be the case in current industrial practice, where other types of mechanical components and tools are produced using subtractive techniques. However, in the near future, in the face of the intensive development of additive technologies, 3D printing might gain an advantage over subtractive technologies, even for complex abrasive tools made of advanced materials and composites. Figure 1a,b show the comparison between the subtractive technique and additive manufacturing technique, respectively, and they differ as follows:

- In AM, material is added layer by layer to build the desired solid 3D product, whereas with subtractive methods, the material is gradually removed from a solid block to fabricate the final parts.
- In AM, complex shapes can be easily fabricated, whereas with subtractive methods, the process has a limited ability to produce complex shapes.
- The AM process is mostly suitable for materials that have low melting points such as polymers. Whereas, with subtractive methods, the process can use all solid materials, irrespective of their melting points.
- AM processes use raw material in forms of powder, filament or liquid; whereas, with subtractive methods, the raw material can be provided as a solid block, forging or cast.
- In AM, volumetric density and the weight of the constructive material of final component can be controlled during operations, whereas with subtractive methods, it is associated with material wastage in the form of chips.
- The AM process can be applied only to a selected range of materials, while subtractive methods can handle a wide variety of materials.

In general, subtractive processes generate more material waste compared to AM processes. In the past few decades, AM has evolved from functional prototypes to exclusively manufacturing various metallic components that cannot be produced easily by other subtractive manufacturing methods [5]. AM technologies allow fabrication of parts with high geometric complexity and reduced post-processing stages [6]. Thermoplastic polymers remain the most popular class of additive manufacturing materials, but the number of metallic materials that are currently being used for metal printing is still increasing. These materials include stainless steels, aluminum, titanium and nickel alloys which are mainly used in laser powder bed fusion (LPBF) and direct energy deposition (DED) [7]. The material feedstock is typically in the form of metal powder or wire being melted using a laser beam, electron beam or an electric arc [8]. Besides that, a component consisting of multiple alloys can also be fabricated by varying different compositions between the layers. Metal printing that is currently used in aerospace, healthcare, consumer products, automotive and other industry sectors has short lead time processes with the ability of using the same equipment to produce different products [9]. Nowadays, metal 3D printing is becoming the fastest growing sector in AM with the viability in diverse industries and the ability to print unique components [10]. However, despite the advantages of metal printing, each printing machine has its own geometrical limitations and complexities. These include the limitations in product size, differentiation of geometrical features and the feedstock materials affecting the overal building components [11]. For LPBF, product size is restricted by the dimensions of the powder bed [12]. Meanwhile, for high deposition rate processes based on fusion welding, there are almost no restrictions regarding the part size as compared to LPBF machines [12]. In addition, the capital investment costs are lower. However, the feature resolution and surface finish are still very poor which may require efforts for further improvements [13]. Moreover, printing with complex design requires support structures which include additional material usage and also post-processing [14]. Complex geometries fabricated by AM technologies comprise of internal features and lattice structures that possess a unique challenge. Therefore, the part design for metal printing involves the consideration of desired geometry, product attributes and manufacturability [12].

**Figure 2:**
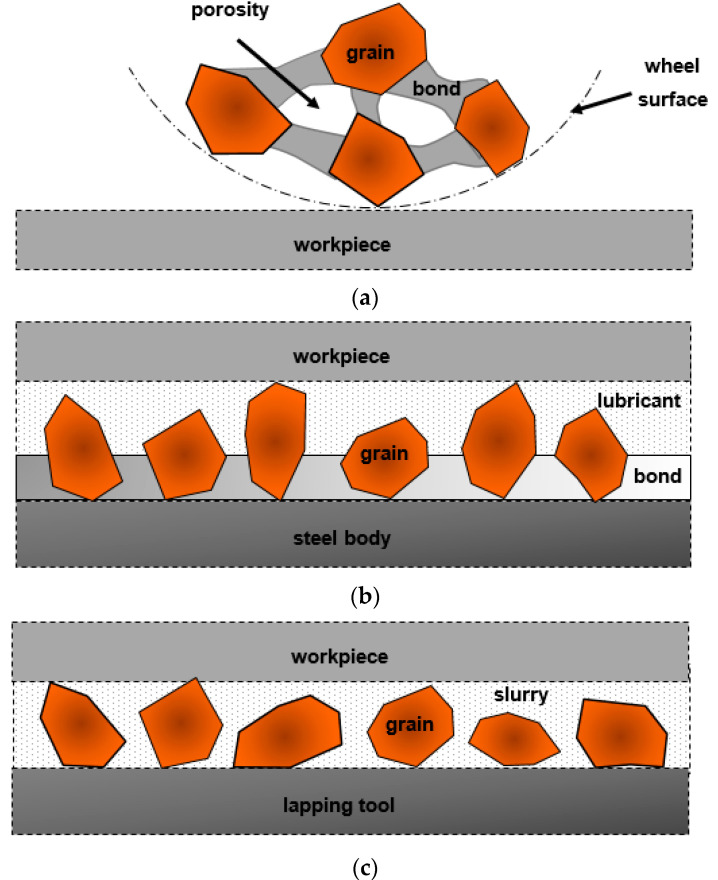
Schematic illustrations of fixed abrasives in a porous grinding wheel (**a**) and an electroplated tool (**b**), as well as loose abrasive used in lapping (**c**).

Works on the development of innovative abrasive tools and methods of their fabrication are the subject of many contemporary inventions. Abrasive processes, such as grinding with the use of fixed-abrasive tools or lapping with the use of loose abrasive are still the most popular and frequent methods applied for parts finishing. These processes are based on material removal from a workpiece through the action of hard, abrasive particles to improve the quality of the parts whether in terms of surface finishing or dimensional accuracy. Bonded abrasives act together as a grinding wheel which can be regarded as an engineered composite material designed for the particular applications and consisting of such elements as abrasives, bonding material and some porosity [15]. Bonding material holds together the grains and some porosity is necessary to provide clearance for the coolant and produced chips—Figure 2a. The performance of grinding tools can be increased by increasing the tool porosity, especially for metal bonded grinding wheels. The structure of electroplated tools is characterized by free spaces between abrasive grains determining free removal of chips during machining, as well as proper delivery of coolant to the working zone [16]. Apart from that, electroplated tools are characterized by high versatility of their applications. These grinding wheels are capable of covering tool bodies of any shape with an abrasive layer, although the internal porous structure cannot be fabricated by electroplating—Figure 2b.

Intensive work on the development of the new constructions of the tool also involves lapping technology. Lapping is characterized by cutting speeds approximately 10 times lower than in grinding, protecting the workpiece from the burns and subsurface damage [17]. Lower cutting speed results in a relatively small amount of the workpiece material removed by loose abrasive suspended in oil or water slurry—Figure 2c. It is used as the basic flattening process to obtain a high degree of flatness of a single surface (single-sided lapping) [18], parallelism between two machined surfaces (double-sided lapping) or sphericity in the case of balls [19]. The machining interaction in grinding is considered to be two-body wear, and that in lapping to be a combination of two-body and three-body wear. The results of work on modifying the shapes and structures of standard lapping plates are shown in [20]. Currently, the work in this area concerns, among other things, the use of non-standard tools containing metallic fiber fillers [21,22,23]. Another example is the development of abrasive tools based on resin and diamond abrasives, which are used in lapping processes without using a standard abrasive slurry—slurry-free lapping [24,25]. In this type of lapping process, abrasive particles can be directly applied to a flat surface of the tool, similar to grinding.

Nowadays, apart from the necessity of obtaining high dimensional and shape accuracy, the efficiency and economic aspects of the process are equally important. A longer effective grinding time and higher material removal rates can be obtained for wheels with controllable abrasive arrangement. To fabricate grinding wheels with a desirable distribution of abrasive grits, different technologies can be used, such as brazing, sintering, electroplating or additive manufacturing. Another crucial aspect of product quality control is the prediction of tool wear which can be optimized on the basis of the uniform distribution of trajectories during abrasive machining with loose [26] or bonded grains [27,28,29].

A review of selected research works shows a strong tendency to develop new designs and fabrication methods of tools used in various abrasive processes that affect the improvement of process efficiency and obtaining satisfactory technological effects. Nowadays, the most suitable methods for making the required internal structures are Additive Manufacturing technologies, but additively fabricated tools for abrasive machining are still under intensive and continuous investigation. Simultaneously, the production of components with complex geometries by additive technologies makes it necessary to adopt finishing processes based on abrasion. Electrochemical polishing (ECP) [30] and ultrasonic cavitation abrasive finishing (UCAF) [31] were used for surface finishing on additively manufactured surfaces. On the other hand, additive manufacturing gives an opportunity to develop new processes and composites, as shown by Hon and Gil in [32] on the example of silicon carbide/polyamide matrix composites produced using the selective laser sintering (SLS) process. Maekawa et al. [33] used the greentape laser sintering (GTLS) method for the fabrication of metal-bonded grinding/polishing tools containing cubic boron nitride (CBN) or alumina abrasives. In the course of polishing austenitic steel with a copper-bonded CBN tool, a mirror surface was achieved. Considering the many advantages of incremental manufacturing over traditional manufacturing [3,34], it can be stated that additive fabrication is a promising but also demanding method for manufacturing innovative abrasive tools, including fabrication with an active means of controlling porosity [35]. Additive Manufacturing technology, described as free manufacturing, creates new possibilities in the development of precisely defined structures of abrasive tools. The examples of additively manufactured abrasive tools described in this paper show their great potential and usefulness in carrying out effective abrasive machining of selected materials, although there are still some limitations for wider implementation of AM of grinding tools on a production scale.

The remainder of the paper is structured as follows: Section 2 of the paper contains the classification of Additive Manufacturing (AM) and post-processing methods. In addition, exemplary application areas of AM are presented. Section 3 presents a review of research performed in the area of abrasive machining with the use of tools fabricated by AM. The possible applications of additively manufactured metal- and resin-bonded abrasive tools are indicated and discussed in Section 4. Finally, the main conclusions resulting from the authors’ research and academic publications are formulated in Section 5.

## 2. Additive Manufacturing

### 2.1. Classification of Additive Manufacturing Methods

Additive Manufacturing (AM), commonly referred to as incremental or 3D printing technology, refers to the layered construction of objects based on 3D computer models with complex inner [36,37] or outer [4] geometries. A 3D computer model is created in CAD software used for computer aided design (CAD) or obtained as a cloud of points by 3D scanning [38]. Simultaneously, AM requires the implementation of several stages, starting from the 3D-CAD model and ending in the physical part. The most important of them are the following stages [39]:

− preparation of a 3D model of the object using CAD software and the creation of its surface model, most often by STL conversion;
− generation of a layered model (slicing process) and setting the process parameters and machine setup, such as the thickness of a single layer of material, energy source, material constraints, printing speed, build orientation, etc.;
− the automated process of building the physical part on a 3D printer;
− post-processing, such as cleaning the physical part from unused material or removing it from the build platform, removing supporting features, carrying out additional treatments to improve the overall appearance of the printed detail and its mechanical properties.

AM technology is a set of methods that differ in terms of several key factors. The most important of these include, among other things, the way of building the object, the type and form of material processed, and the source of energy generated for the incremental process [40]. The selection of a specific 3D printing method, together with the type of building material and a set of process parameters, affects the obtained mechanical and physical properties of the detail, which also determines its future application. Another equally important element is the class of the applied AM printing system. The current development of 3D printing methods is therefore closely related to the processing of new types of construction materials, as well as the use of high-performance 3D printers [41,42].

The classification of AM methods can be based on different criteria. Table 1 shows the most popular division of AM methods according to the type and form of the building material used. Additionally, the main types of methods and leading manufacturers of 3D printers were indicated for each group [43,44].

Generally, according to ASTM 52910, there are seven types of AM technology: photo polymerization, material jetting, binder jetting, material extrusion, powder bed fusion, sheet lamination and direct energy deposition, where the first four are among the most popular technologies used in this area. Powder bed fusion uses a high power-density laser to melt and fuse metallic or plastic powders together [45]. Examples of technology using this technique include selective laser sintering (SLS) and laser powder bed fusion (LPBF). According to ISO standards ASTM 52900-15, laser powder bed fusion (LPBF) is also known as selective laser melting (SLM), direct metal laser sintering (DMLS) and laser metal fusion (LMF) [46]. The other technique is material extrusion, where filament material is extruded from the high-temperature nozzle. This is the most popular technique selected by users, especially hobbyists, because it is available with low-cost 3D printers. As for vat polymerization and material jetting, both use liquid resin for the material. These technologies produce higher accuracy and smoother surface finishing compared to others [34].

The working space of the chambers limits the size of parts produced by the additive technologies discussed so far. Additive technologies originating from fusion and solid-state welding expand the potential of AM. Wire arc additive manufacturing (WAAM) can be used to produce medium-to-large-sized thin-walled parts at lower cost [47]. WAAM adopts arc welding tools and wire as feedstock for the fabrication of structures of low to medium complexity [48]. Although the parts built by WAAM are near-net shape, they often show significant anisotropy in terms of both microstructure and mechanical properties [49], with crack-like defects formed under unfavorable deposition conditions [50]. This is mainly due to the fact that in fusion-based AM technologies, the temperature involved in the process is higher than the melting point of the materials to be joined. Among the various arc processes that can be used for wire melting, the cold metal transfer (CMT) process seems to be one of the most suitable for WAAM, thanks to its controlled current waveform and filler wire feeding, which allow regular deposited weld beads to be obtained [51].

In solid-state AM processes, the temperature involved is lower than the melting point of the materials to be joined, preventing the defects occurring in fusion-based AM processes. In addition, the main challenge in production of dissimilar material structures relates to the formation of different brittle intermetallic compounds at the interface as a result of different coefficients of thermal expansion and different crystallographic structures [52]. When lower temperatures are involved in the AM process, dissimilar material structures can be fabricated without generating harmful intermetallic compounds [53]. Additive Friction Stir-Deposition (AFS-D) is a solid-state AM process derived from friction stir welding, preventing hot cracking by avoiding liquid–solid phase transformations [54]. Friction surfacing for layer-by-layer manufacture of three-dimensional metallic parts can be realized in both single- and multi-track approaches, with excellent bonding between individual layers and tracks [55].

Low-pressure plasma spray and cold spray deposition, traditionally used for obtaining solid-state surface coatings, also have high potential to be used as additive manufacturing technologies. In cold gas dynamic spray (CGDS), a wide range of metallic and non-metallic materials can be successfully deposited onto a substrate material to ensure corrosion protection and increase mechanical durability as well as wear resistance. This process can be used not only to manufacture thick coatings but also freestanding parts or to repair damaged components [56,57].

### 2.2. Post-Processing Methods in Additive Manufacturing

As was mentioned previously, several attempts have been made to overcome the challenges in maintaining acceptable surface quality and mechanical properties. These include controlling various process parameters [58] and applying post-processing techniques on AM fabricated parts, to name but a few. It is known that the major problem of AM printed parts is caused by staircase effects [59]. Staircase effects vary depending on the selection of process parameters, for example, layer thickness. Lower layer thickness values produce smoother finishing; however, selecting lower values of layer thickness alone will not dissolve and completely diminish the staircase effects, and still high values of surface roughness may result from machining [60]. In certain applications, where smooth surface finishing is required, for instance, to resist corrosion, post-processing is found to be the most practical method. Generally, post-processing techniques in AM can be divided into two categories: mechanical finishing and chemical finishing.

Mechanical finishing, concentrated on mechanical cutting or pressing, is used to alter the surface of a manufactured item to achieve a certain quality values, such as appearance and adhesion. This technique is similar to conventional metal finishing techniques; however, it has been found that this method produces compelling results in plastic in comparison with metal [61]. Early findings for post-processing methods using mechanical finishing in AM were presented by Spencer et al. [62], who investigated two automated finishing techniques known as vibratory bowl abrasion finishing and ultrasonic abrasion finishing. These two techniques produced an acceptable surface roughness for SLA parts. It was concluded that the vibratory bowl abrasion finishing process produced a very good surface finish, with improvements of 74% from the actual Ra value (surface roughness measurements) compared with other methods. In contrast, material extrusion techniques using the FDM method produce a relatively low surface finish, with the Ra parameter being between 9–40 µm [63]. Various post-processing methods including manual sanding, abrasive milling, abrasive flow machining, sand blasting, vibratory bowl finishing and hot cutter machining have been suggested to solve these issues.

Sand blasting uses a sandblaster, tools for finishing with a powder coating, or paint. Sand blasting has been found to be an impressive method, improving the surface finishing of the printed components by up to 96% [61]; however, the finishing process can be more difficult for products with complex shapes. Therefore, several modifications have been reported, but these still possess several limitations. For example, implementing hand polishing, such as manual sanding, would increase the labor cost, time and poor surface quality due to the application of inconsistent skill. Laser polishing, chemical polishing, electrochemical polishing and abrasive machining have shown great potential for AM surface treatment. In powder bed fusion, for instance, laser polishing uses a direct laser source in the laser melting deposition equipment, but this technique may cause thermal damage [64].

**Figure 3:**
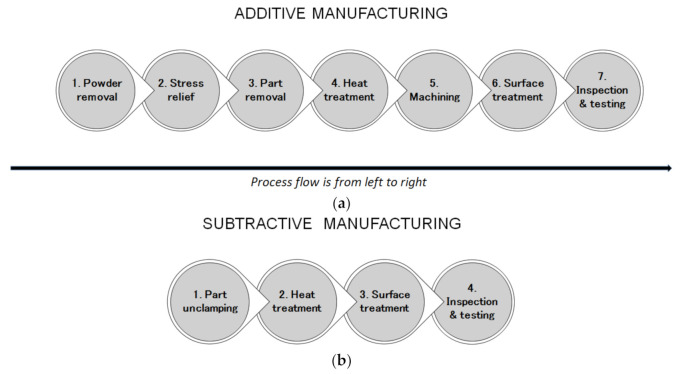
Possible post-processing steps for parts fabricated additively (**a**) and using material removal processes (**b**).

Chemical finishing, such as dipping the parts in acetone vapor and blowing cold acetone vapor [65], can be used to effectively improve surface finishing in FDM parts by 99%. However, this procedure may raise environmental, health and safety concerns. In addition, chemical polishing can be effectively used for complex surfaces, such as lattice and porous structures [66]. Furthermore, abrasive machining has been found to be an effective process for polishing metal-based AM parts with complex internal cavity and channel structures, and this method is widely known as abrasive flow machining [67]. Figure 3 shows the possible post-processing steps for parts manufactured additively using laser powder bed fusion (LPBF) and parts manufactured using material removal processes.

In metal printing, a part is basically ‘welded’ on the top of a building platform, and it is usually impossible to pull the part off without any assistance. Even after removing the part, post-processing needs to be conducted before the part is completely made for functional purposes. Overall, there are seven steps in the post-processing technique for AM, starting with the process of powder removal. AM parts are built ‘deeply’ in a powder bed fusion system, and new layers are added to the top, which means that the parts are buried in powder when the printing process is completed. The heating and cooling of the metal when the part is being built in a layer-by-layer manner lead to internal stresses that need to be relieved through a stress relief process before the part is removed from the building plate. Otherwise, the part may warp or even break before it can be removed from the plate. Stress relief requires equipment such as an oven or furnace that is able to fit the entire build plate. After part removal, heat treatment, such as aging and annealing [68], needs to be conducted in order to improve the microstructure and mechanical properties of the part [69]. The next stage is the machining process, e.g., making holes and threads and ensuring the necessary dimensional and shape accuracy of the part has been achieved. Additional surface treatment can be conducted to improve the surface finish. This also includes cleaning the internal channels or removing the partially melted particles on the part [70]. During the final stage, inspection on the basis of contact or non-contact measurements, as well as non-destructive testing (NDT) [71], is usually conducted.

In Figure 3b, the post-processing steps for subtractive manufacturing are presented. There are usually fewer steps in post-processing for subtractive manufacturing, but significantly more machining operations, tools and machine tools are required for the whole production process in comparison to AM technologies—Figure 1. Generally, AM technologies are characterized by material savings and shorter lead times, which are particularly useful advantages for low-volume production, but the required post-processing steps must be carefully considered at the design stage.

### 2.3. Exemplary Application Areas of Additive Manufacturing

Another aspect of 3D printing technology often addressed in the literature is possible areas of application for printed elements. The authors of the papers [72,73] indicate, in this respect, three main areas of 3D printing technology:

− Rapid Prototyping, which, as the name suggests, concerns the production of prototypes of future objects. The area of rapid prototyping, due to the significantly limited possibilities of the primary 3D printing methods, has been the basic application of this type of technology. At the same time, despite the dynamic development and capabilities of modern AM methods and systems, rapid prototyping is still one of its key areas of application. Therefore, the term Rapid Prototyping is very often used to comprehensively define all 3D printing methods. Moreover, making a prototype of a future object and already checking its properties at the design stage results in a significant reduction of costs connected, among other things, with possible product errors;
− Rapid Tooling, which is related to the rapid prototyping or manufacture of tools or elements of production equipment. Currently, AM methods play an important role in the production of both the tooling used in traditional forming and casting methods (Indirect Tooling) and the direct production of tools and production tooling elements (Direct Tooling/Prototype Tooling);
− Rapid Manufacturing, indicating the possibility of building objects with features similar to those obtained using traditional subtractive or plastic forming methods. The abovementioned dynamic development of AM printing methods, the materials used, and modern AM systems is favorable to the achievement of better and better functional properties of printed elements. Currently, mainly AM powder methods with metals and their alloys are used to build fully functional and responsible mechanical parts. Elements of this type are used in the aerospace, offshore, and energy sectors [36,74,75] as well as in the automotive, electronics, medical [76,77] and tool industries [78,79], among others.

**Figure 4:**
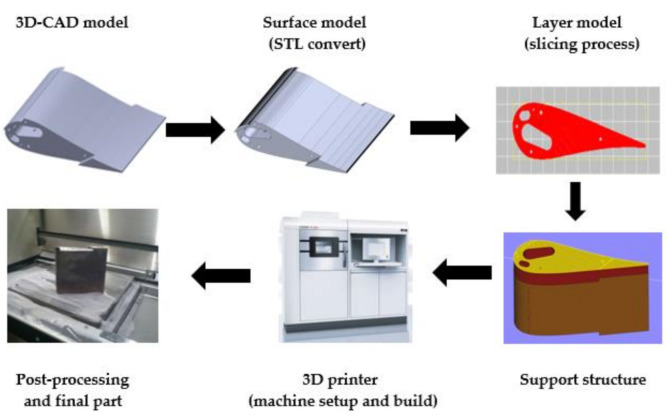
3D printing process chain for the example of a turbine blade produced by means of DMLS technique with the use of EOS Maraging Steel MS1 in the form of powder; based on references [36,37].

The example of the 3D printing process chain of a turbine blade is illustrated in Figure 4 [80]. After building a 3D model, the part was manufactured using EOSINT M280 3D printer (EOS GmbH, Krailling, Germany) with the thickness of a single layer of laid material of 40 μm and a total number of model material layers of 3834. One of the essential structural elements of the presented blade, which needs to operate under high-temperature conditions, is the proper design of its inner features, ensuring cooling medium flow and effective separation of blade material from hot gases. The results presented in [36] indicated that the sintering process did not make it possible to make the holes of the diameter equal to 0.3 mm with the angular position. This revealed the limitations in application of selective laser melting technology with respect to making holes with a diameter smaller than 1 mm. This also indicates the limits to manufacturing inner structures with small dimensions. The measurements performed with the use of computer tomography and digital radiography confirmed that the DMLS technology made it possible to directly produce holes 3 mm, 2.5 mm, 2 mm, 1.35 mm and 1 mm in diameter under different angles during additive processing. As the prediction of hole clearance is a crucial task when designing the complex internal geometries in mechanical components, Deja and Zieliński [37] proposed an in-process inspection method for the evaluation of the dimensional and shape accuracy of straight through holes made of AlSi10Mg and MS1 test samples manufactured using DMLS technology. The experiments performed enabled the development of a general mathematical model for determining the hole clearance for the given sample thickness *G* ∈ [1,5] and the theoretical hole diameter *d_t_* ∈ [0.3, 1.5]. This gives some suggestions for the achievable dimensions of inner features. The general tendency observed for all the tests, and confirmed by qualitative evaluation, indicated that reducing the hole diameter and length increased several material defects which, along with structural changes, affected the differences between the theoretical (assumed) and determined diameters. The material discontinuities inside the holes, characterized by the lack of the full material melting, confirmed the difficulties in making small-diameter features, especially in thin plates. This disadvantage of AM may influence the AM fabrication of abrasive tools requiring special inner structure or features characterized by small dimensions.

## 3. Application of AM Technologies in Abrasive Machining 

The review of current research works indicates the growing importance of AM technology in the area of abrasive machining, mainly in the production of prototype constructions of grinding wheels, as well as tools used in lapping and polishing. Metal-based AM methods represented by Powder Bed Fusion, such as DMLS (Direct Metal Laser Sintering) and SLM (Selective Laser Melting) have been successfully used in the production of grinding wheels. Grinding technology is one of the most important abrasive process in traditional manufacturing. Previous research [81] indicated the modified grinding wheel designs as one of the main objectives in the contemporary development of grinding technology. The research mainly concerns the production of tools with increased porosity and expected internal structures. Due to the limitations of conventional hot pressing sintering fabrication process, many researchers focus on the use of innovative materials and novel techniques to produce grinding wheels, including current laser process technology [82,83]. One of the examples is a 3D controllable abrasive arrangement grinding wheels which enable to achieve high machining performance [29,84].

### 3.1. Metal-Bonded Abrasive Tools

As presented in [35,85], metal-based 3D printing methods, such as SLM process, were successfully used to fabricate the metal-bonded diamond tools. Regular distribution of grains can improve surface roughness of machined surfaces, tool life and grinding efficiency. The application of incremental manufacturing enables the control of abrasive grain distribution in multiple layers and in three dimensions as shown by Yang et al. in [86]. The authors developed a method based on the AM technology by adopting CNC laser machine for the production of end face diamond wheels with regularly placed diamond grains in the metal matrix. Grinding experiments confirmed the aplicability of printed tools characterised by the wear typical for wheels with super-hard abrasive. Micro- and macro-fracturing of grains created new cutting edges improving the grinding performance. Laser cladding is another succesfully introduced method of manufacturing textured CBN/CuSnTi grinding wheels resulting in lower grinding forces and temperature in comparision with electroplated grinding wheels [83].

Denkena et al. [87] used the laser powder bed fusion (LPBF) technique to fabricate NiTi diamond composites. Although LPBF is a technique for a large range of metal powders, these types of specimens for use as grinding tools were manufactured for the first time. Their usability was proven in scratch tests confirming that diamond grains were firmly embedded in the bond. The authors concluded that LPBF process can be classified as suitable for the production of abrasive tools used to grind tungsten carbide material. Another important direction pointed out by authors for the further research on metal-bonded wheels was that LPBF process parameters for pure metals are not completely transferable to metal diamond composites characterized by the lower density of energy necessary to produce the specimens.

Diamond blade segments with a 3D lattice of diamond grits were additively manufactured on a production scale amounting to 216,000 pcs/month, using a new type of AM equipment with a rotary working platform [88]. Needle jig with inner holes and negative pressure was used to ensure the repeatable arrangement of diamond grits in certain directions and not by using the powder mixing. The tool containing additively manufactured diamond segments exhibited better grinding performance in comparison to conventional blades of equal thickness with randomly distributed diamond grits at the same concentration of grits. In addition, self-sharpening characteristics was observed due to new cutting edges revealed during the cutting time.

**Figure 5:**
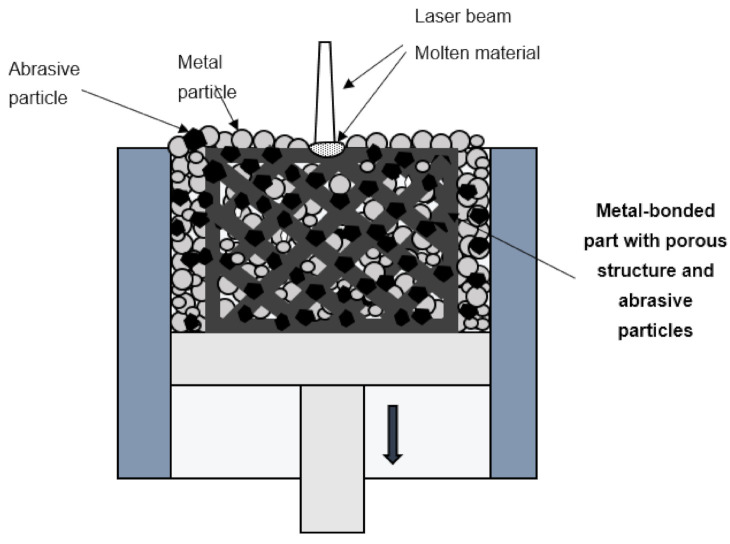
The principle of the SLM process for the fabrication of a grinding wheel with the structure of high porosity; based on references [91,92].

The abovementioned methods have many advantages with respect to grinding efficiency, but the surface finish has not been studied in detail by the authors. The required texture of added bonding layers and determined 3D-arrangement of super hard abrasives were controlled during these laser-based fabrication methods. Nevertheless, it was impossible to control the internal structure of the tools as porous metal-bonded grinding wheels have several advantages compared with dense metal-bonded tools. Other metal-based AM methods have been widely used to fabricate porous tools as reported in [89,90,91]. Characteristic pores in the grinding wheel lead to greater space for the transport of lubricant, coolant, and chips. AM technologies may reduce the number of defects and can be more effective in controlling the final porosity and micro-structure in the comparison to conventional manufacturing methods of grinding wheels with the application of, e.g., pore inducers. In general, wheel fabrication by SLM technology is based on the method for manufacturing metal matrix composites, in which the composite consists of metal matrix (binder) and inclusions (abrasives)—Figure 5. Tian et al. [92] obtained the optimal SLM process parameters for manufacturing metal matrix composite with abrasive grains, i.e., laser power of 250 W, scanning speed of 2.5 m/s and layer thickness of 20 µm, in terms of comprehensive mechanical properties and final surface finish. Moreover, microscopic observations showed firm embedding of uniformly distributed diamond abrasive particles (size 65–75 µm) into aluminum alloy binder (AlSi10Mg) with few void defects detected. In addition, some manufacturing limits, typical for SLM and DLMS methods, were formulated such as a minimum thin wall thickness of 0.3 mm, a minimum circular hole diameter of 0.2 mm, and a minimum square hole side length of 0.3 mm. These limits could be the indications for design and fabrication of customized grinding wheel with controlled porosity or micro channels. Other restrictions pointed out by the authors as limiting the industrial applications of SLM-fabricated grinding wheels were related to the size of abrasives and wheels, although the SLM process is very suitable to produce controlled metal structures of high porosity.

Different cellular structures with the same porosity of 53% were tested in [90], showing that the largest strength and permeability was achieved for octahedron structure of the metal matrix with abrasive. The diamond abrasive grains were uniformly mixed with the AlSi10Mg alloy powder at the concentration of 60%. The applied values of laser power (300 W) and layer thickness (30 µm) were slightly higher than the values used during experiments presented in [92]. In general, diamond abrasive grains did not melt during the SLM process, but the microscopic observations revealed some burnt grains due to the huge energy density of laser, resulting in surface graphitization of diamond [90]. Such defects do not result in the desired interfacial reaction between abrasive particles and the metal bond but decrease the holding strength of abrasives in the metal matrix. When optimal process parameters were used, the grain-bond interface properties studied in [90,91] showed that both carbon and aluminum elements with certain contents existed at the transition region between grain and bond, forming the chemical metallurgical bonding at the grain-bond interface. This kind of the interfacial reaction between abrasive particles and the metal matrix increases the holding strength of abrasives for grinding wheels. A similar positive effect was observed in [86], where Cr-C compound was found on the interface of the diamond grains, strengthening the degree of binding between the metal binder and the diamond. In addition, grain concentration and distribution in metal matrix influence strongly the mechanical properties of a grinding wheel. As shown in [93], grain clusters with uneven grain distribution formed weak points in the grinding layer.

The effectiveness of the proposed method for printing metal-bonded grinding wheels was confirmed by the grinding experiments presented in [35]. A grinding wheel with a honeycomb porous structure was used to perform the grinding tests on the Cr4W2MoV workpiece. The obtained results indicated the good cutting ability of the tool with firmly bonded diamond abrasives in the metal matrix. The average abrasive pull-out rate of abrasive grains was 17.2%. The greatest number of results obtained during grinding with the use of tools fabricated by SLM was presented in [89]. Three kinds of grinding wheels, including octahedron, honeycomb and solid structures, were tested, showing excellent dressing and self-sharpening characteristics. The surface roughness was not sensitive to different structures, grinding time or grinding depth, but the solid structure wheel featured the largest material removal rate.

Advantages and disadvantages of abrasive tools fabricated by LPBF processes and conventional manufacturing methods, such as sintering, electroplating and monolayer brazing, that have been used in industry so far are presented in Table 2. It can be clearly seen that in terms of most comparative criteria, tools fabricated by conventional manufacturing methods show considerable advantages over additively fabricated tools. Taking into account the criteria of geometric complexity as well as cost and efficiency of manufacturing, additive fabrication of tools is becoming more competitive, especially for tools with strictly defined internal structures and features but with limited values of achievable dimensions.

### 3.2. Resin-Bonded Abrasive Tools

**Figure 6:**
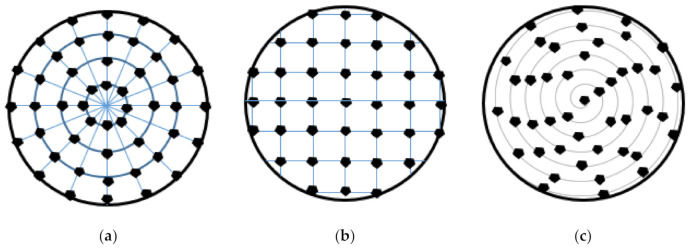
Schematic illustration of circular (**a**), rectangular (**b**) and spiral (**c**) patterns of abrasive grains arrangement in X-Y plane of grinding wheels studied in [29] and made by a stereolithography process.

Additive manufacturing technologies have been effectively used in manufacturing of resin-bonded grinding wheels. Qui and Huang [29] fabricated tools with 3D controllable arrangement of abrasive (Figure 6) using a developed stereolithography (SLA) apparatus equipment based on the additive manufacturing technology. Similarly as for the metal-bonded grinding wheels with regular grains distribution, wheels produced by the SLA method with regular abrasive distribution were more effective at material removal in comparison to wheels with random arrangement of abrasives. In addition, the uniformity of grinding trajectories can be optimized at the design stage of the tool by determining the required abrasive spatial arrangement.

**Figure 7:**
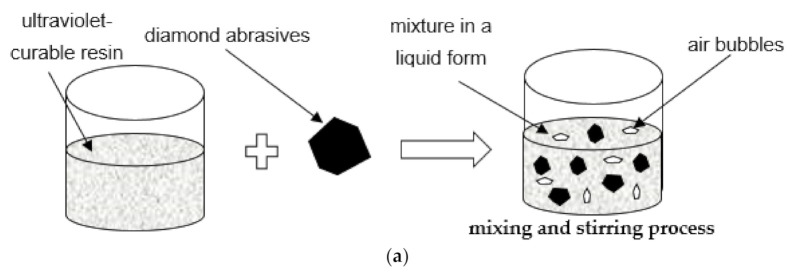

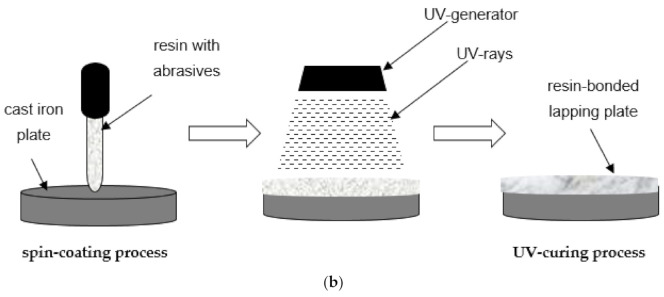
The fabrication process of a resin-bonded lapping plate: (**a**) preparation of mixed ultraviolet-curable resin and diamond abrasives; (**b**) UV-curing process with the use of spin-coating method; based on references [95,97].

Another example is the production of abrasive tools in the form of grinding [94] and lapping wheels [95,96] from UV-curing resins. In this method, UV-curing resin and diamond abrasives are mixed together through a stirring machine and applied to the top surface of the base plate followed by UV-ray curing—Figure 7. Simultaneously, as presented in the paper [97], the liquid mixture can be spin-coated directly onto the base plate or injected into a separate fan-shape mold. Compared with the conventional sintering process, this method of fabrication of the resin-bonded tools saves energy cost, as well as labor effort. Nowadays, resin-bonded diamond abrasive tools prepared by this method, are widely used in the precision grinding or lapping of hard and brittle materials, such as technical ceramics. Application of resin lapping plates containing diamond abrasive grains in the machining of ceramic materials allowed obtaining a lower average value of roughness parameter Ra [97] and higher material removal rate [98] compared to the conventional slurry-based lapping process with a cast iron plate. The experimental results presented in [97] showed a significant improvement in surface roughness and reduction in material removal rate during the lapping of a ceramic workpiece and with the use of a resin-bonded tool. In comparison with iron plate slurry lapping, an approximately 10% lower surface roughness was achieved. Simultaneously, about 25% less material removed per minute from ceramic workpiece was observed. Although the authors of the abovementioned studies did not use the term additive manufacturing, the phenomenon of photopolymerization of resins is used in several 3D printing methods, including Stereolithography SLA, Poly/Multi-Jet Modeling, Digital Light Processing and Film Transfer Imagine, among others. Abrasive-mixed resin diamond bond grinding and lapping wheels and polishing discs produced by SLA-based processes were investigated in [94,99]. SLA, as one of the basic and widely recognized pioneer 3D printing technologies, is based on selective curing a polymer resin layer-by-layer with the use of a UV laser beam. Tanaka and Isono [94] pointed out the influence of exposure time of UV light on the depth and width of the cured resin. It was found that only 5–10 s exposure time was enough to produce a small area of the prototyping tool. Huang et al. [99] studied the impact of abrasive diamond concentration on the mechanical properties. It was concluded that the depth of cured resin and mechanical properties decrease with the concentration of the abrasive grains. Simultaneously, with high concentrations of abrasive grains, a higher increment of hardness was observed. Additionally, the authors investigated the influence of three different exposure times of 40, 60 and 80 s, in the performance of the UV-bonded wheel during machining of ceramic workpieces. The best surface quality was obtained for the tool with 80 s exposure. Moreover, SLA-printed lapping tools can be also used as loose abrasive finishing of glass workpieces. Williams [100] analyzed the impact of layer thickness in the performance of lapping plates manufactured by means of SLA. The use of a single layer thickness of 50 µm compared with 100 µm of resin allowed much more efficient removal of material from the glass workpiece. As presented in [101], the 3D printed method of Film Transfer Imagine, also based on UV-curing resin, was used to quickly produce a lapping disk with strictly defined geometry, including the pattern of grooves located on its active surface. However, these UV-curing resin abrasive tools had several limitations, such as high tool wear and relatively short tool life. As reported in [100], the wear of the SLA printed lapping plate during glass machining was much greater than the amount of the removed material. Consequently, their applicability is limited only to finishing prototype operations. Other aspects in the case of resin-bonded abrasive tools containing diamond grains are the control of process parameters of their fabrication, a uniform distribution of the grains into the resin agent as well as the appropriate conditioning method.

One of the most promising AM techniques in recent years is selective laser sintering (SLS), where the particles of a polymer powder are selectively sintered by a laser beam. As presented in [102], this method was used in the production of components with high mechanical properties and complex structures, such as porous polyamide mold for pressure slip casting. Du et al. [103] proposed the SLS method for manufacturing resin bond diamond grinding wheels with internal cooling holes. The results from grinding glass indicated that the grinding force decreases with increasing cooling hole diameter. Additionally, larger cooling holes increase the supply of the coolant and lubricant in the grinding zone and accordingly reduce the friction between the abrasives and the workpiece. Consequently, due to the improvement in the cooling and lubrication effect, wheels containing internal cooling holes reduced the grinding forces.

## 4. Discussion

Grinding, lapping and polishing are the basic manufacturing techniques used for obtaining fine surface finish along with high dimensional and shape accuracy through reduction of surface roughness and precise removal of material [17]. Research in grinding attempts to enhance economic and ecological properties and performance to extend grinding applications in the overall process chain—on the one hand, in the direction of increased material removal rate, avoiding turning and milling and, on the other hand, in the direction of fine finishing, thus making further abrasive finishing processes such as lapping and polishing obsolete [81]. The possibility of further exploring various process parameters in order to produce abrasive tools using additive technologies may result in a more optimal solution using an AM fabrication process compared to traditional methods. Even though the use of AM technologies is quite common, the actual challenge of using the powder-fused process, which lies in its extrinsic and intrinsic properties, to yield desired performance characteristic needs to be resolved [104]. Metal AM processes are relatively slow and expensive, but new types of machinery allow additive fabrication of metal-bonded abrasive diamond tools even on a production scale amounting to hundreds of thousands pcs/month [88]. The knowledge of the optimal process parameters for the fabrication of polyamide or metal matrix composites along with Design for Additive Manufacturing (DfAM) tools and techniques [105] can be a key issue for wider implementation of AM in the production of grinding tools. Apart from the achievable dimensions of some features such as thin walls or holes [37,92], an effective control of the tool porosity and its micro-structure are the main advantages of AM processes.

This review and analysis of selected scientific papers clearly shows that incremental methods may play a crucial role in the further development of abrasive tools. They mainly concern the fabrication of prototype abrasive tools. Due to the requirements and feasible fabrication methods, abrasive tools used in grinding and lapping can be divided into three main categories, with appropriate 3D printing technologies being given in Table 3. Selected characteristics of materials and their compositions used to manufacture grinding wheels by AM technologies are presented in Table 4.

Nowadays, powder-bed methods involving metal and plastic are most widely used in the production of abrasive tools dedicated to grinding operations. As summarized in Table 3, they are used to fabricate tools containing abrasive grains and tools with strictly defined external and internal structure. On the other hand, as was presented in [106], surface finishing is one of the crucial challenges of metal-based 3D printed parts. For example, the surface roughness of SLM printed elements achieves the value of Ra in the range of 10–50 µm. Other difficulties are associated with effective machining of parts with internal complex geometries, including metal-bonded abrasive tools. Currently, some non-conventional finishing techniques are proposed to improve the surface quality of 3D printed metal parts, like abrasive flow machining (AFM) and ultrasonic abrasive polishing [64]. Considering the high surface roughness resulting from powder-based AM techniques, much more attention should be paid to studying the influence of abrasive tool surfaces on the obtained technological effects, including the quality of the workpiece surfaces. For this reason, wider experimental tests should be performed using different abrasive tools.

In lapping technology, softer tools enable effective embedding and exposing of the abrasive grains during machining. For this reason, resin-bonded tools have been tested by many authors. Resin-bonded abrasive tools enable low surface roughness and relatively high process efficiency to be obtained, even when machining hard and brittle materials like ceramics. On the other hand, due to the high tool wear, the time of their effective processing is significantly reduced.

Despite the great potential of 3D printing technologies, abrasive tools fabricated using conventional manufacturing techniques are still the most commonly used in industrial practice. One of the crucial factors determining this situation is their ability to work under variable and heavy loading conditions, as well as machining a wide variety of materials. An analysis of the selected research works clearly shows that the AlSi10Mg alloy powder is widely used as a binder for metal-bonded grinding wheels fabricated by LPBF. It allows the proper holding of unmelted diamond abrasive grains. Resin-bonded abrasive tools are mainly used as lapping or polishing discs, as well as grinding wheels. Their fabrication process is generally based on mixing UV-cured resin in liquid form with abrasive grains. Tools fabricated by SLS have not been widely investigated so far, and this may be a new direction for further research work. Considering the characteristics of SLS printed parts, the tools fabricated by this technology could be applied in various abrasive processes, but with rather light loading conditions. 

## 5. Conclusions

This review of current scientific works shows the growing importance and great potential of 3D printed abrasive tools. As pointed out in several studies discussed in this paper, metal powder technologies can be used as potential methods to produce high-performance novel grinding wheels. The opportunity to produce internal structures in the form of pores and channels of any geometry has many positive consequences. These concern both the machining process and its economic aspects. In the case of lapping technology, many studies deal with the development of UV-curable resin bond diamond plates. The photopolymerization process is characteristic for many 3D printing methods, such as Stereolithography SLA or Film Transfer Imagine. Furthermore, it is possible to print resin lapping plates with shapes that are difficult, and in some cases even impossible, to manufacture using traditional casting or material removal methods. In addition, resin lapping wheels containing abrasive grains allow a two-body abrasive process to be carried out, similar to that during grinding. This enables higher efficiency of machining in comparison with the standard slurry-based lapping (three-body abrasive process). On the other hand, resin abrasive tools are characterized by high wear and relatively short life-cycle time, especially during the processing of hard materials, like technical oxide ceramics. This significantly limits their wider industrial applications. Nevertheless, taking into account such an intensive pace of development of AM technology, there is a chance for it to become one of the key methods for in the production of novel and high-performance abrasive tools. The results of the authors’ research accompanied by academic publications allow the formulation of the following conclusions:

- Additive manufacturing has great potential in the manufacturing of abrasive tools used especially in finishing processes. In the near future, in the face of the intensive development of additive technologies, additive manufacturing could be competitive with conventional technologies used for the fabrication of abrasive tools.
- Anisotropic mechanical properties of the 3D printed components are one of the basic disadvantages of the additive technology. This may affect the proper functioning and operational safety, especially when metal-bonded wheels work under variable and heavy loading conditions.
- The component orientation in the working chamber of a 3D printer should be carefully analyzed at the design stage, as it influences the final structure and, as a consequence, the strength of the fabricated elements.
- Metal-bonded wheels made by AM technologies are characterized by high efficiency, but the surface finish after grinding using these tools has not been studied in detail by researchers.
- For geometrical features of smaller dimensions, more material defects, i.e., the material discontinuities characterized by the lack of the full material melting, as well as higher inaccuracy, may result from the production of tools using AM technologies. The difficulties in making small-diameter features may influence the AM fabrication of some abrasive tools requiring specific and controlled inner structures.
- So far, AM-based methods have mainly been dedicated to the fabrication of prototype abrasive tools. LPBF technology is the most promising method for the fabrication of metal-bonded abrasive tools, whereas UV-based processes, including SLA technology, are the most promising for resin-bonded tools. The number of tests and experiments performed with additively manufactured tools is still very limited, which makes them difficult to apply on a large scale in industry.

## Figures and Tables

**Table 1:** An overview of various AM methods.

Name of a Group	Powder Bed Fusion	Material Extrusion	Vat Polymerization	Material Jetting
Material form	powder	filament	liquid resin	liquid resin
The way of building the object	sintering or melting of plastic or metal powders with laser or electron beams	extrusion of material through printheads and onto a build platform	exposure of a photopolymer resin to the light and its polymerization	spraying a photopolymer resin from tiny nozzles in a printhead and its cured using UV light
Technology (short name)	SLS; LPBF; EBM; MJF	FDM/FFF	SLA; DLP; CDLP	PJM; MJM
Common manufacturers	EOS GmbH; SLM Solutions; 3D Systems; Concept Laser; Arcam	Stratasys; Ultimaker; MakerBot; XYZ Printing; Prusa Research	3D Systems, Formlabs, MoonRay	3D Systems, Stratasys

Footnotes: SLS—selective laser sintering; LPBF—laser powder bed fusion; EBM—electron beam melting; MJF—multi jet fusion; FDM/FFF—fused deposition modeling/fused filament fabrication; SLA—stereolithography; DLP—direct light processing; CDLP—continuous direct light processing; PJM—polyjet modeling; MJM—multijet modeling; EOS—electro optical systems.

**Table 2:** Comparison analysis of LPBF and conventional manufacturing processes used for the fabrication of abrasive tools.

Comparative Criteria	LPBF Processes	Conventional Manufacturing Methods
Mechanical properties and shape accuracy	Low, due to the process characteristics and the anisotropic mechanical properties which may affect the proper functioning and operational safety under variable and heavy loading conditions;	High, enabling the proper functioning and operational safety under variable and heavy loading conditions
Defects	Occurring frequently, e.g., balling phenomenon, micro-cracks, voids, distortion, delamination, material discontinuities inside the internal small features; difficult to correct during post-processing; graphitisation of diamond particles	Occurring rarely, e.g., micro-cracks, inner cavities, local breakouts, tool unbalance
Microstructure	Sintered or melted metal matrix with regular or irregular arrangement of abrasive grains of limited type and size	Wide variety of abrasive grains of different types and sizes, bonded to the binder and randomly oriented in the space
Geometric complexity	High, with strictly defined inner shapes, including the internal porous structures and other features, like cooling holes and channels with limited values of achievable dimensions	Low, with significant limitations in the fabrication of complex geometries, particularly internal structures, including pores
Fabrication costand efficiency	Cost-effective solution for unit and small batch production, dedicated mainly for the fabrication of prototype tools; a very limited number of tools fabricated on a production scale	Cost-effective solution for series and mass production; high variety of tools which can be used for finishing different mechanical components, including parts of complex shape
Fabrication process	Simple, with small number of required devices for the fabrication and post-processing	Complex, with various manufacturing machines and equipment required for the fabrication
Technological effects obtained after machining with abrasive tools	High efficiency and fine surface finish obtained in experiments reported in the literature; very limited experimental data due to the small number of tested tools, especially in industrial conditions	High process efficiency, particularly during high speed grinding; fine surface finish of mechanical components made of different materials

**Table 3:** Abrasive tools fabricated using incremental methods.

Type of Abrasive Tool	Type and Form of Building Material	Fabrication Method	Characteristics of the Abrasive Tool	Applications in Finishing Operations
metal-bonded	metal matrix composites consisting of a metal matrix (binder) in powder form and inclusions (abrasive grains)	LPBF	regular or irregular arrangement of abrasive grains in the metal matrix; controlled porosity and defined internal structure	Grinding, polishing [33,35,85,86,87,89,90,91,92]
resin-bonded	a mixture of ultraviolet-curable resin in liquid form and abrasive grains	SLA; film transfer methods; other methods based on UV-curing resin	regular or irregular distribution of abrasive; strictly defined geometry, different patterns of abrasive grains arrangement;	Grinding, lapping, polishing [29,94,95,96,97,98,99,100,101]
powder-bed fusion	sintering a plastic material in powder form	SLS	tools with strictly defined internal structures with cooling holes and channels	Grinding [103]

**Table 4:** Materials and their compositions used to manufacture grinding wheels using AM technologies.

	Type and Form of Binder	Abrasive Material	Mixing Ratio between Binder and Grinding Material	AM Fabrication Process
metal-bonded tools	AlSi10Mg alloy powder [92]	diamond	diamond abrasive grains (particle size: 65–75 µm) and AlSi10Mg alloy powders (particle size: 15–53 µm); lack of information about mixing ratio	LPBF
	AlSi10Mg alloy powder (model: BH AlSi10Mg) [35]	diamond: GMD650	diamond abrasive grains (particle size: 65–75 µm) with 15% volume fraction and AlSi10Mg alloy powders (particle size: 15–53 µm) with 85% volume fraction	LPBF
	AlSi10Mg alloy powder [89]	diamond	diamond abrasive grains (particle size: 62–75 µm) with 15% volume fraction and AlSi10Mg alloy powders (particle size: 15–53 µm) with 85% volume fraction	LPBF
	AlSi10Mg alloy powder [90]	diamond	grain concentration (vol.%)—60	LPBF
resin-bonded tool	acrylate UV-cured resin in liquid form [94]	alumina: WA #1000	grain concentration (vol.%)—25 [others: curing agent (wt.%)—5]	SLA or other methods based on UV-curing resin
powder-bed fusion tool	nylon PA2200 in powder form [103]	diamond: W40	nylon (wt.%)—67.5 diamond concentration (vol.%)—12.5 [others: glass bubble (wt.%)—20]	SLS
			nylon (wt.%)—67.5 diamond concentration (vol.%)—12.5 [others: white corundum (wt.%)—20]	

## Data Availability

Data sharing not applicable to this article.
